# The Hospital. Nursing Section

**Published:** 1904-08-27

**Authors:** 


### The Hospital.

### Nursing Section.

Contributions for this Section of "The Hospital" should be addressed to the Editor, "The Hospital" Nursing Section, 28 & 29 Southampton Street, Strand, London, W.C.

No. 935.—Vol. XXXVI. *SATURDAY, AUGUST* 27, 1904.

### Notes on News from the Nursing World.

### OUR CHRISTMAS DISTRIBUTION.

As usual at this period of the year we remind our readers of our Christmas distribution of useful articles of clothing for the patients in hospitals and infirmaries. We make an appeal thus early, while yet the summer-holiday season is in progress, because it may be easier for some who desire to help us in this annual labour of love to spare time to make the kind of garments which are most acceptable. Moreover, our experience last year, when we drew attention to the matter in August, was so successful in enlisting immediate responses that it justifies us in repeating the experiment. Parcels may be sent, directly they are ready, addressed to the Editor, 28 & 29 Southampton Street, Strand, London, W.C., with "Clothing Distribution" marked on the outside.

### WAR OFFICE RECOGNITION.

The War Office authorities desire to communicate with Miss M. C. Bakkes, Mrs. Rutherford, and Miss M. B. Horswell with reference to the services rendered by them as nurses in the Boer Refugee Camps in South Africa during 1901-1903. Any information as to the present addresses of these ladies should, it is added, be forwarded to the Secretary, War Office, Pall Mall, S.W.

### THE MISSING NURSING ORDER.

Parliament has been scattered to the four winds of heaven, and doubtless the President of the Local Government Board is enjoying his holiday. But before Mr. Long left London he omitted to issue the order which has been awaited ever since the report of the Departmental Committee on Nursing was submitted to him. It is impossible to suppose that he has not fully considered the recommendations of the Committee, and we are driven to the conclusion that he either intends to treat the report as waste-paper, or cannot make up his mind what course to pursue. Meanwhile, there is a revival of the complaint that Poor-law nurses are treated by voluntary hospitals as inferior to hospital nurses, and that the tendency to exclusiveness is extending to the larger workhouse infirmaries themselves. We need only say now on this point that nurses holding the certificate of the recognised schools of training among Poor-law infirmaries ought to have free and open egress from the service into other departments of nursing, and that any attempt to bar their way is opposed to the true nursing spirit. But Poor-law nurses and the public also have a right to know definitely whether the President of the Local Government Board intends to act upon any portion of the report of the committee he appointed, or to permit things to remain as they are.

### STATE REGISTRATION.

A single fact in connection with the proceedings of the Select Committee of the House of Commons on Nursing suffices to show the absurdity of the suggestion that the Committee should have issued a report. The number of witnesses examined was eight. What possible significance could attach to any conclusions based on such utterly inadequate evidence as this? The most exhaustive investigation, and the examination of witnesses of the most representative character, without axes to grind, is essential to the publication of a report of the slightest value.

### THE MIDWIFERY QUESTION IN NEW ZEALAND.

In a memorandum issued by the Premier of New Zealand on "Child Life Preservation in New Zealand," Mr. Seddon advocates that a measure for the registration of midwives should be passed on the lines of our own Act of 1902, absolutely prohibiting any but registered midwives to practise in 1906. The need of the measure in New Zealand is clearly shown by the return which was recently prepared at the instance of Mr. Seddon. It extends over ten years, and during that period 20,000 children between the ages of one and five died—a rate of mortality which is out of all proportion to the population. Mr. Seddon is on much more debatable ground when he proceeds to urge that the State should train nurses in the hospitals for the purpose of attending on the sick poor. In England, of course, the State provides nurses for the poor in the workhouses, but this is a very different matter from training them to nurse the poor in their own homes.

### A QUESTION FOR THE LOCAL GOVERNMENT BOARD.

The Hampstead Guardians, instead of, like the Scarborough Guardians, making a grant on their own account to the medical officer for his services in instructing the probationers, have submitted the question to the Local Government Board. Subject to the sanction of the Board they have decided to award him £20, and £10 to the superintendent of nurses. These are fairly liberal grants, but if the Local Government Board do not object, no one else is likely to take exception to them. It is, however, most desirable, not to say essential, that the sanction of the Local Government Board should be obtained before the money is paid in such cases.

### FUNERAL OF NURSE RILEY.

The funeral of Miss Elise Riley, whose death under singularly sad circumstances was reported last week, took place at the parish church, Stroud, on Friday afternoon. At the inquest a verdict of "Accidentally drowned" was returned, the jury joining with the coroner in an expression of sympathy with the relatives of the deceased. Miss Riley was regarded at Guy's Hospital as a good nurse, and she was much liked both by the authorities and by her fellow-nurses.

### NEW RULES AT ST. ANDREW'S HOUSE CLUB.

In another column will be found a letter from a member of the St. Andrew's House Club, who is evidently very dissatisfied with the manner in which that institution is managed. It is only fair to remind her that she need not belong to the club if she does not like either the charges, the accommodation, or the rules. The proprietress in the circular which she has issued to nurses, enclosing a copy of the new rules, expresses her regret that, owing to the rents, rates, taxes, and general expenses of the club having considerably increased since it was opened four years ago, she is obliged to make a slight increase in some of the charges, notably in that for bedrooms, of which only a few will be let in the future at 2s. 6d. a night. Boarding terms are to be altogether abandoned. It is added that there are a few alterations and additions to the club rules, and we think that the proprietress has done all that can be expected from her in earnestly asking nurses to note the changes and additions. As they do not come into force until September 1st, we do not see that there is any question of a personal grievance.

### POSTCARDS FOR PENSION FUND NURSES.

We are asked to draw the attention of Pension Fund Nurses to the offer made by The Scientific Press to supply them with postcards bearing photographs of scenes at the Royal Reception at Buckingham Palace, which they can procure for one shilling and sixpence a dozen, or twopence for a single card.

### POOR-LAW NURSES AND PRIVATE CASES.

An important question has been discussed by the Guardians of the Banbridge Union. It appears that complaints have been made to the Irish Local Government Board that the superintendent nurse attends private patients outside the workhouse hospital. The Board having asked the Guardians for an explanation, several of the latter expressed the opinion at their last meeting that there was no objection to the superintendent nurse assisting the workhouse doctor at operations on outside patients. We agree with the Guardians who take exception to this opinion. The services of the superintendent nurse of a workhouse infirmary whose salary is paid by the ratepayers should certainly not spend any of her time in attendance on cases outside the institution to which she is attached. The regulations of the Irish Local Government Board do not, we believe, allow the Guardians to exercise any discretion in this matter.

### APPEAL BY THE MATRON OF A LEPER ASYLUM.

Miss A. M. Whiteman, matron of the Pretoria Leper Asylum, who is now in England, appeals for a few extra comforts and amusements for the unfortunate people in the institution with which she is connected. As she explains, all the necessaries of life are found by the Government, and her anxiety is to do everything she can to relieve the monotony of the isolated existence which the poor lepers are compelled to lead. She particularly desires to take back to South Africa a magic lantern, with a good supply of slides; footstools, invalid chairs, pipes, tobacco-pouches, pictures, picture-books, games, music, and toys. The patients, some 300 in all, are of varying ages, from tiny babies to elderly men and women, 45 of them being white persons. Gifts in kind, strongly packed for export, should be addressed to Miss Whiteman, care of Messrs. Bullard, King, and Co., East India Docks, London, each package being marked "Presents for Lepers at Pretoria"; and any sums in money for the purchase of articles to the Hon. Lady Lyttelton, Stocks Cottage, Tring. We hope that some of our readers may be able to assist in this admirable movement.

### THE NURSES OF SUNDERLAND INFIRMARY.

An interesting feature at the annual meeting of the Governors of the Sunderland Infirmary this week, Lord Durham in the chair, was the presentation, after the despatch of the usual business, of silver medals and certificates to the nurses. Medals were awarded to Nurse F. E. Saville, for Hygiène, and Nurse M. Ferguson, for Hygiene and Physiology; and certificates were given to Nurses Davidson-Woodhouse and Lawrence on completing their three years' training. Great interest was manifested in the proceedings by a large number of citizens, and before the meeting, which was held in the grounds of the infirmary, the guests were entertained at tea by Mr. and Mrs. Stansfield Richardson.

### A CHARMING MODE OF HELPING QUEEN'S NURSES.

Bazaars, fancy fairs, concerts, and football matches in aid of nursing institutions are all to be welcomed, but the hon. sec. of the Inverness branch of the Queen's Jubilee Institute, Miss Mackintosh, of Raigmore, hit upon the happy idea of a flower show and competition, which accordingly took place the other day in the drill hall of the 1st V. B. Cameron Highlanders at Farraline Park. The competing tables of flowers were got up with admirable taste and harmony of colours. The first was an arrangement in pale yellow, with brown and green foliage, and the second consisted of blue sweet-peas and other flowers of the same shade of colour. The combinations included pink geraniums on a centre of silver gauze, over green smilax; blue and pink peas with green trailers; pink poppies with sweet-pea centres and yellow grasses, and purple heather and ferns. There was also a competition of cookery and dairy produce, but the flower show was quite the feature of the exhibition, and we are not surprised at its popularity.

### SHORT ITEMS.

Wolverhampton Workhouse Infirmary has been certified by the Local Government Board to be a training school for nurses.—The parish of Nottingham *Nurses' League Journal* for August, which is a highly creditable production, shows that the number of members—most of them nurses at the Infirmary, Baythorpe—is upwards of eighty.—Seven probationers of the Sunderland Union Infirmary have passed the examination of the London Obstetrical Society. They received their instruction from Miss Pruett, superintendent nurse.

### The Nursing Outlook.

**"From magnanimity, all fear above;**

**From nobler recompense, above applause,**

**Which owes to man's short outlook all its charm."**

### THE NURSE AS HEROINE.

Some further additions to the "Roll of Honour" have been suggested to us since our article under that heading appeared last week; and again the stories are of all ages, and all countries, and all creeds.

In the eleventh song of the *Iliad* we are told of Mulios, whose daughter, Apanede the Fair, "understood the healing nature of all the herbs which grew upon the earth." Let us take it for granted she was nurse as well as druggist. The healing art was not minutely divided up in those days. Another Greek girl, Nikarete, who was very rich, and who lived in the time of Chrysostomus, took a house in the poorest part of Constantinople and went and lived there and nursed the sick, and compounded drugs for them free of charge. Then there is our own Queen Matilda, wife of Henry I., and sister of St. David of Scotland. She brought the "Poor Clares" or "Sisters Minor" to England, and established them in the Minories; and close by she put the hospital of St. Giles-in-the-East, for the maintenance of 40 lepers—quite the most important leper hospital in England. Her pity seems to have been specially for those afflicted with this disease, for Ailred of Rievaux tells how she brought the lepers into her own royal apartments, girded herself with a towel, and then washed them.

Let us turn to the domestic servant for our next heroine. Monna Tessa was a housekeeper to an old gentleman at Florence, and she worried him dreadfully by bringing sick folk into his house to nurse: he was always finding them hidden away in corners! So at last he bought her the house next door to put her sick folk in, and when he died he left her his money, and she founded the big, beautiful hospital which is in use to this day.

Our Irish readers will not forgive us if we leave out St. Bridget, who was always running about nursing the sick. There is a delightful story of how two lepers came to her one day, and she set them to wash one another. When the first had washed the second, the second became clean and whole of his disease. Bridget was charmed, and bade him now wash his brother. Not a bit of it! He turned away, saying a whole man could not touch a leper. "Then *I* will wash him," said St. Bridget, and did so; and, when washed, the second leper became clean and whole and remained so; but meanwhile the first man's leprosy reappeared, and he went wailing away into the night!

Of special interest at this time is the history of Mme. Bakounina, known as the Russian "Florence Nightingale." At the time of the Crimean war she was head of the Russian order of sisters of mercy, and attended operations at the field hospitals regardless of danger. She and her sisters also had the courage to discard their uniform and don sheepskin coats and long boots and go out in the snow and render first aid, and help bring in the wounded. This order of nurses, which was founded by the Grand Duchess Helena Pavlovna in 1854, has many members now at the front, and keeps its jubilee in October. Then we must not forget that most modest and most heroic of women, Mary Kingsley, who laid down her life at Cape Town whilst nursing the Boers of typhoid. The niece of Charles Kingsley, she was a writer and traveller, and ever in her wanderings she ministered to the sick. Witness some of the stories told in her delightful "Travels in West Africa." At the time of the Boer War she slipped quietly away to Cape Colony to do whatsoever her hand should find to do for those who were ill. Most of her friends never knew she had left England till they heard of her death. She took the most unpopular work and the hardest work. Not hers to rush to the front with the soldiers and the doctors and the clamour and excitement. She nursed the Boers on the typhoid hospital ship till she herself took the disease and died. And there were so many others we could have better spared!

Another heroine of our own times is described in a French volume just issued called *En Haut!* The Countess de Saint Martial was left a widow in 1885, and she put aside her position and wealth and joined the Order of St. Vincent de Paul. She nursed and organised and toiled for them in France and Italy till, after a brief 11 years, she died worn out. Her published letters prove her to have been not only a good nurse and a clever woman, but also a good Catholic.

Then there is Sister Trina, head of the Protestant deaconesses at Reihen, near Basle, whose labours and influence were described in a paper written by Agnes Jones. The principal sphere of work of the deaconesses is a hospital, so unpopular at the time Sister Trina took charge of it that the patients had to be bribed to enter it! Miss Jones described Sister Trina as "a simple and humble-minded Christian, with gentle, winning, and affectionate manners," and elsewhere she mentions her "beautiful humility." And yet the deaconesses under her not only nursed the hospital, but had charge of a deaf institute, a children's hospital in Basle, did private and district work in Basle, and worked in Basle gaol. And these sisters were chiefly of the peasant class. If we have not mentioned Agnes Jones herself, or Sister Dora, or other of our great English heroines, it is because full memorials of their lives have been published, and ought to be in all nursing libraries and familiar to all nurses.

### Lectures Upon the Nursing of Mental Diseases.

By Robert Jones, M.D.Lond., B.S., F.R.C.S.Eng., M.R.C.P.Lond., Resident Physician and Superintendent of the London County Asylum, Claybury.

### LECTURE XVII.

The duties of the private nurse in charge of mental cases are more onerous and of wider extent than fall to the lot of those who nurse the insane in institutions. She may have to nurse cases not only in their own homes, but possibly in lodgings, and she will not be able to summon to her aid all those helpful means which have steadily grown up in the best private and public institutions, and which constitute the great advantages of such places as curative establishments. The private nurse may probably have to select the sick-room for her patient, and she may be called upon to do a considerable amount of sick nursing, and it is important, as was made clear in the first lecture, if she is to render real help, that she should be well trained in hospital as well as asylum nursing. There are large numbers of mentally affected persons among the wealthy classes who are cared for outside asylums, and many also in asylums who have, in the earlier stages, received home treatment before being certified for detention in hospitals and asylums. The trend of general opinion at the present day is to try the nursing of all early mental cases in private homes, under specially trained nurses—*i.e.* before sending them for treatment into asylums—and a Bill at present before Parliament has for its sole object the legalising of such treatment in private houses which is now in England and Wales contrary to law. The class of cases which it is intended should be nursed in private homes is from those whose mind is weakened either from old age, or as the result of long continued epilepsy, or those suffering from gross lesions of the brain—such as cerebral tumours, cerebral hæmorrhage, or embolism; cases of hemiplegia in whom the power of speech, active locomotion and supervision of the calls of nature, are impaired; cases of congenital deficiency, imbecility or idiocy and terminal cases of paralytic dementia. Speaking generally, cases with marked excitement, or those who are suicidal or who exhibit dangerous propensities are not treated in their own homes, but they are certified for detention in hospitals, licensed houses (otherwise called private asylums), or public asylums, unless they are cases of mental excitement in young persons or pregnant women or those whose insanity is accompanied with puerperal conditions or even those who are suffering from the temporary maniacal conditions caused by alcohol. All these can be suitably treated for a time in private houses under the care of private nurses. Should the present Bill, which is now before Parliament become law, which will legalise the treatment of "incipient insanity" in private houses, a great need will then arise for properly-trained mental nurses and the following observations, in addition to those referring to nurses in institutions, may be found useful.

It must, at the outset be understood that no amount of training and no amount of special experience, whether in a hospital or an asylum, can replace the judgment and common sense which a nurse needs who is directly engaged with the insane. She must be her own key of interpretation as to what should best be done in any special emergency, and her own native tact will prove more helpful to her than any amount of fanciful theories. The school of experience associated with sound training will make the best nurse, as it will make the best surgeon and the most practical physician. The old-fashioned asylum nurse—who probably could neither read nor write, but who possessed a clear head—has more often relieved anxiety, saved risks, and been of more comfort to patients and their friends than the brilliant probationer; indeed, more helpful than the newly fledged degree holder who has just emerged from hospital with his yet untried medical qualifications, and whose practical work among men and women has yet to be learnt and acquired. I do not undervalue brilliant attainments, both in the case of nurses as well as doctors; such are not to be decried, for they are useful handmaidens, and will without doubt assist those who possess them, but they will not serve the place of those qualities which are inborn and which cannot be artificially raised, viz., tact, patience, devotion, good temper, sympathy, sound judgment, and an alert mind—characters which are absolutely essential to the good mental nurse, whether engaged in public institutions or in private work. The private nurse is more the companion of her patient than is the ordinary institution nurse, she may have to read, play, or sing, she may have to "maid" her patient, and she will certainly have to nurse her.

As to the sick-room, it should have, if possible, a southern aspect, so as to allow of the possibility of sunshine when the weather does not permit of exercise, or when from other causes the patient is compelled to remain indoors. If possible, it should be spacious, lofty, and well-ventilated, as the habits of the insane are often such that the atmosphere may soon be rendered close, oppressive or offensive, and may thus injure the health of both nurse and patient. The room should be made cheerful and pleasant to the eye, with wall-paper of not too demonstrative a pattern, and there should be bright flowers and pictures about. To avoid risks in consequence of stairs the rooms for a mental case should if possible, be on the ground floor. It is advantageous, however, in case of noise or outburst of excitement on the part of the patient that the apartment should be somewhat isolated, and it should be away from avenues of traffic or causes such as may disturb the rest or sleep of an irritable, nervous or excited sufferer. A natural prospect with a good view of field or garden is preferable to that of a thoroughfare with its various disturbances. There should be a second room opening out of this and a bath-room and lavatory with all the necessary adjuncts, which should have perfect drainage. It so happens that such ideal arrangements are possible for the private nursing of mental cases, as many of these cases can afford to pay for all that is needed. It is already provided for the poor who cannot so pay, in the palatial homes created by the various county authorities and met with all over the country.

In regard to ventilation, the purity of the atmosphere is of the highest importance, and this can best be secured by natural means, *i.e.* by open windows and open fires. In the case of many insane persons the emanations from the surface of the body and from breathing soon vitiate the air, and when these emanations are combined with dust and other impurities, the air, unless frequently changed, is soon rendered unfit for respiration. Fresh air should be admitted without draught, and for this purpose it is best to keep the window open at the top. An open fire is one of the best means of diffusing currents of fresh air through a room and fresh air should be obtained from the outside and not from passages or rooms in the house. The temperature should be kept at 60°, or higher for old persons, and it should not be allowed to fall below 50°. The windows of rooms in which insane persons reside should only open about five inches above and below to avoid danger from escape, homicidal, or suicidal attempts. The glass should be guarded in the lower pane or panes by an iron screen of a light and artistic

### LECTURES UPON THE NURSING OF MENTAL DISEASES—*Continued.*

pattern, so as not to obtrude the idea of seclusion. The furniture should be of a simple kind, strong and comfortable. There should be no keys left in the locks for the patient to isolate herself. There should be no cords to the blinds and no picture wires or aught that could suggest self-injury to a depressed or suicidal patient. The carpets should not cover the whole room, and matting can often be substituted for rugs or mats. The bed and bedding should be of a simple form. The male or female nurse usually sleeps in the same room with a private patient of the same sex unless the case be very acute. The bedstead should be placed where light cannot fall on the patient's face in the early morning, and the light should be at the back, not in front. A horsehair bed on a spring mattress is quite satisfactory, and the bed linen should be clean, light and smoothly folded. Comfortable pillows—care must be taken to avoid feather pillows for epileptics—must be arranged, and if the patient is feeble or bedridden a bed-rest to support the shoulders will afford much comfort and it will be a change for the patient to be occasionally propped up. An eider-down quilt is always preferable to heavy bed-clothes, both from a health and comfort standpoint. If the patient be a chronic case, feeble or paralysed, she may have to be kept in bed for long periods; in such cases an air or water-bed or cushion is a necessity, for it equalises the pressure and helps to prevent bedsores. Bedsores are, in some cases, a flagrant example of bad nursing; when present they are most difficult to heal, and their prevention is always better than their cure. I admit that in some cases where there are great nutritional disturbances, bedsores or pressure sores will inevitably occur, but a general test of good nursing is their absence. The patient should be kept scrupulously clean, changed whenever necessary and at once when the occasion arises, not at fixed intervals. The skin of the back should be frequently examined and washed clean with soap and water, then with methylated spirit, brandy or eau de Cologne, dried carefully and afterwards powdered with violet powder, starch, or boracic acid powder. I have known a female patient who had to be, together with her bedding, carefully changed not less frequently than 17 times in a day. A "mackintosh" sheet is placed under the lower blanket in cases needing special supervision, viz., such as those whose habits are defective, and when not in use this should be rolled and not folded.

The nurse herself should wear a quiet and unobtrusive uniform, as she seems to be more in authority when wearing a special dress, and there will be less risk of personal antagonism when she is thus attired. The uniform should be of some light, washable material, and of a colour that will not readily show the dirt. A print dress is always a useful indoor uniform, as it can so easily be washed. A neat cap, apron, and cuffs complete the toilet. The nurse should have a good pair of scissors for emergencies, attached to a chain and kept in a special shielded case. The patient's face and hands should be washed before each meal, at least three times a day, and the body sponged with tepid water whenever necessary. The teeth should be regularly cleaned, and any artificial teeth taken out at night, cleaned, and placed in a tumbler of cold water till the morning, when they are handed to the patient or placed by the nurse in the mouth. The hair should be carefully tended to; combed, brushed, and then plaited into neat braids. The nurse for private mental patients, as stated, must be maid, companion, nurse, and friend.

### The Private Nurse: Her Duties and How to Perform Them.

BY A PHYSICIAN.

MUCH that is valuable and interesting has been written on the subject of hospital nursing, but the literature relating to private nursing is scanty. It is with a view to supplying what is lacking that I have been induced to write this short paper. Personally I am of opinion that the successful hospital nurse, as a rule, succeeds equally well when she is brought face to face with a private case. At the same time I have known not a few nurses who did well while under training, and yet failed hopelessly when they left their hospital wards. In order to make what I have to say as short and yet as clear as possible, I shall state the private nurse's duties under three heads. As soon as a nurse enters upon the arduous duties of nursing a private case she finds herself placed in relation to three very different parties. First there is the patient himself, then there are his relatives, and last but not least his medical attendant has to be considered.

### The Patient.

First then as regards the patient. His position in life will probably be quite different from that of the ordinary hospital patient. The nurse must not forget this. At the very outset then she must show him due respect. She is for the time being his servant, paid to attend to his wants, and the nurse who is too high-minded to remember this fact will never prove successful. She may have spoken sharply to some hospital patient when he did something wrong, but any attempt on the nurse's part to "snub" a private patient will at once be ruinous to her prospects. She must be quiet and unassuming in the sick-room. Very frequently patients are extremely sensitive to the slightest noise. The creaking of a chair, the rustling of a dress, or the turning over of the leaves of a book may disturb them. The nurse should, therefore, move about the room as little as possible. There should be no justification for the complaint that just as the patient was falling off to sleep the nurse woke him up. The nurse who understands invalid cookery is one who is sure to be much sought after. Especially she should know how to prepare eggs in different ways, how to make custards and various beverages, and to cook fish, and to make such things as beef tea and chicken soup not only palatable but really presentable to the patient. When convalescence is being established she may be asked to read to the patient. The nurse should therefore cultivate the art of reading aloud in a clear and yet soft voice. She should have a supply of topics upon which to converse with patients in different ranks of society. On no account should she weary them with details of interesting cases she has seen during her hospital days. These may interest her, but they rarely do an invalid. What he wants is pleasant and bright conversation. Try to find out his pet subject or hobby, and keep talking about it. By so doing you will rise in your patient's estimation. Do not forget to shake up and turn his pillows from time to time. Nothing is so comforting to an invalid as to feel the head resting upon a cool, fresh surface.

### The Patient's Relatives.

Then there are the patients' relatives—so often dreaded, and perhaps justly so, by the private nurse. This is really the great stumbling-block in the way of many a nurse's ultimate success. Relatives usually vote the nurse a nuisance, though withal a necessary one. She upsets their household arrangements to some extent, and they dislike her for it. Then they are apt to interfere with her in her duties. They suggest this, that, and the other thing. I am talking now, of course, only of those cases where the relatives are fussy. They will constantly come into the room when the patient has just fallen asleep and wake him up. Then in the matter of ordinary cooking they are often ignorant. The medical attendant may order a certain thing and the cook makes it ready according to her own ideas, the result being often far from satisfactory. If the nurse suggests that she herself should prepare any special dish, she is thought to be interfering. As a rule, however, where the food is not such as is likely to suit the requirements of the patient, the nurse should never hesitate to tell the friends plainly, but of course in as kindly a manner as possible, that she will be glad to help them with the extra cooking that has to be done. A wise nurse will never express any opinion about the patient, good or bad, to his relatives. They may ask her if she thinks he is better or worse, but she should not allow herself to be led into an expression of opinion which may turn out to be quite erroneous. Her replies to any such inquiry should always be most carefully guarded. She will often be asked about her own creature comforts, such as what food she prefers. She should not hesitate to speak out her mind. It is wise, however, not to ask for out of the way things or for articles of diet which are difficult to procure. Some people will appear to be willing to give the nurse anything she asks for, but unfortunately such people often complain that the nurse is very exacting in her requirements. The nurse who can adapt herself to her environment as regards food is certainly to be envied, as this goes a long way towards winning the admiration of the patient's relatives.

### The Medical Attendant.

Finally, she has the medical attendant to consider. Nurses, do be careful how you take the temperature and pulse. Cultivate exactness in these matters. How often have I seen a subnormal temperature recorded when in reality it should have been raised. The average thermometer should be kept in position for at least five minutes. This fact should be written and rewritten time after time upon the nurse's memory. Personally, I have little confidence in the nurse when I find the temperature badly taken. Do not be afraid to tell the medical attendant if anything has gone wrong since his last visit. The good nurse is one who keeps nothing back. If you can help the medical attendant by giving him any useful information by all means do so. If the patient cannot take the medicine ordered, or finds the bandages too tight, say so. No one will think less of you for speaking out. Talking of medicines, never allow lotion bottles to be placed alongside of the patient's medicines. I constantly see this done by nurses in private, and I often wonder why they do so, seeing that in hospitals there is always a special cupboard provided for poisons. While the medical attendant is examining the patient the nurse should be at his side, ready to anticipate his wants. She should know by experience what she requires to do to aid him in his examination. If he requires a lotion, the nurse should have it ready in an instant. He may consider it necessary to give a hypodermic, she should have water and whatever else may be required at hand. Then when everything is over, and the medical man wishes to cleanse his hands, the nurse should have hot water, soap, and towel in readiness just as if they had been lying waiting all the time he was in the room. The nurse who can arrange all these matters to a nicety is sure to succeed. After all, the power of adaptation to circumstances is the key to success as a private nurse. She must be bright and cheerful, firm and calm in every emergency, hopeful of the patient's recovery even when he is being despaired of, and above all attentive to his every want. The comfort of her patient must be her first thought. To be approved of by his relatives should occupy her attention next, while throughout and pervading all her work she should endeavour to carry out the wishes of the medical attendant. The nurse who excels in private work is not always the most brilliant, but she is the one who is looked on with favour alike by patient, friends, and doctor. The latter sings her praises when he tells the friends "that's a good nurse."

### Odd Leaves.

BY A SISTER.

An old note-book, nearly dropping to pieces with wear, recording the cases of a busy ward for five years. It is not much to look at, chiefly names and dates, with a line or two for diagnosis, and a very small column for "results," and here and there a tiny copy of an unusual temperature chart, but it is illustrated through and through to the eye of the owner with mental pictures of the patients represented by the "cases."

### Memories of Tears.

Here is a page opened at random, "Charles Stevens, van-boy, age fourteen, Jan. 25 to Feb. 11." A stunted youth rises in our mind's eye, who has the appearance of not having washed since Sunday morning at latest, and of having tossed about in pain the whole night through. "Pain?" he should rather think so, with many grunts. "Last meal?" Ten o'clock last night. "Viands?" Nuffink to hurt, a bit of cold pork and a pennorth of winkles. "And so to bed," as old Pepys would say, and a very harassing time for Master Stevens in divers respects for the whole day through. He was better the next morning and tolerably cheerful, till the visiting physician had washed his hands and departed. Then he was found with his head under the clothes sobbing for all he was worth. An offer of a fresh hot flannel soothed him not (almost infallible remedy though it is!) nor a hot drink, nor even a suggestion that his mother would be up to-morrow. But at last it was sobbed out that the old gent had said he'd got the purisy, and he knew he should die. "A chap that worked along o' me had purisy, and he died, they always do." Poor little van-boys! they suffer a good deal with their chests, for they are constantly exposed to the weather, and work very, very long hours, especially at Christmas and other holiday times; and yet one never hears of any such efforts for their happiness and well-being as are made for so many other classes of the King's subjects, this Coronation year. However, friend Charlie went out alive and happy, labelled "Colica ab ingesta. Pleurisy c effusion."

There is another memory of tears a little further down the same page. A labouring man, unmarried, well over fifty, was discovered mopping his eyes with a very wet handkerchief on the evening of his arrival. No, he had no pain—hadn't heard any bad news—was not uncomfortable—but he had never left home before, and he couldn't help crying.

### Strange Diagnoses.

New patients are apt to volunteer rather strange diagnoses of their own complaints as every nurse knows. Here is one. F—— S——, 27, chronic nephritis, had been in hospital before. "What was the matter with you then?" "They said it was Hysteria, Miss," was the reply, given with considerable pride. But the poor man was dead in a week;

ODD LEAVES—*Continued.*

another example of what experienced nurses have often noticed, that there is not infrequently a good deal of the "neurotic" element in patients with any form of kidney disease. H—— B—— came in attended by his son, a sharp Board School boy of twelve. He also told the tale of having been in hospital a year ago, but before he could answer the usual question of "What was it last time?" the boy cut in with the reply "Natural causes, sister!" probably a reminiscence of the verdict in some coroner's inquest he had attended.

DODGING THE INSPECTOR.

Turn over a page or two. Here is "Tom Griffiths, 7, schoolboy." But he must have dodged the inspector pretty successfully to judge by his remarkable want of civilisation. He was a typhoid, brought up to the hospital in an ambulance, and for the first day or two of his sojourn he spent all his waking and non-feeding hours in wailing, "Send for the fever cart to take me 'ome! O, send for the fever cart to take me 'ome!" After a few weeks of a sharp attack we thought we might send him there, not in an ambulance, but by way of the convalescent home, and we began to talk cheerfully to Tommy about clothes for the great occasion, though with concealed doubts as to their suitability, and on next visiting day he was found in a state of almost speechless indignation. "Hi, nurse!"—we could never break him of that form of address—"My little sister's bin and sold my clothes at the rag shop?" "What did they give her for them?" "Five fardens, nurse!" And we all thought they must have been sold at full value.

A BIT OF SUNSHINE.

On an earlier page comes the record of James Sullivan, aet four, a delightful, never-to-be-forgotten little Irishman, and a real bit of sunshine in the ward. We used to think he might make a great novelist some day, his inventive powers were so amazing. He would tell endless yarns about "our court," and his daddy's great deeds, especially with the "coppers," of whom Jimmy did not stand in any awe whatever, by his own account. Indeed, he used to represent himself as being on familiar terms with these great men. Sometimes we wished that Jimmy would have a little more regard to the truth. "Where is Sister"? the doctor asked him one day when he came for an unexpected visit at an odd time. Jimmy always knew, or thought he did. "Gone to get her half pint," he piped out. And naturally the doctor did not let sister forget where she had been that afternoon. Jimmy had a funny, touching little faith in the Unseen. He possessed a favourite doll, which had come badly to grief. He was rather disconsolate, till he bethought him of what to do. "I shall take her up to Heaven," said Jimmy, "and I shall say to God, God, mend this gal's 'ead!" He had his ups and downs in the ward for three months, when one bitter January day his mother appeared in great tribulation. His father had sent her to bring the child home, and had threatened her with most awful menaces if she did not fetch him. So our boy had to go, with a sad little face, for the remembrance of a miserable home was evidently strong even in his childish memory. A month afterwards a great friend of his, whom he always called "my nice doctor," to distinguish him from "the tall doctor" and "the ginger doctor" (though they were all equally devoted to Jimmy) went down to the wretched court where his mother lived to inquire for him. A slatternly woman lounging in a doorway answered, "Jimmy Sullivan? Oh, he was buried last week."

CURIOUS PREFERENCES.

"Alfred Jones, 8, enteric," is chiefly remembered for a quite unusual liking which he had, while he was still on milk diet and getting very hungry, for watching his neighbours feed. "Pull back that screen please, sister," he used to say, "I likes to see them have their dinner. Leave the kitchen door open, so I can see your hands carving. I know they's your hands, they's so red!" Poor sister! One somehow remembers the children better than the older patients; now and then with sad memories. There was one little lad of ten who had a difficult appetite and a taste for extraordinary articles of food—a kipper, liver, stewed eels, etc.—and one day no one had any peace until he was provided with "the roe of some fishes." He was irritable and exacting beyond all words, and seemed to spend all his time in inventing work for the nurses. And then he would look at one sitting down by his bed to rest her tired legs a minute, and would say complacently, "I'm sure *I* don't give you much trouble, nurse!"

A PERFECT LITTLE GENTLEMAN.

Another boy, suffering from the same form of heart complaint, was, though he belonged to a drunken father and a disreputable mother, as perfect a little gentleman as could be found in London. We used to call him "Mr. White," a title he would accept with a pleased little smile. He was a frequent visitor in the ward for many months, and was always the same quiet, uncomplaining little patient. The last time he came in was from a "home" in the country where he had hardly been happier than in his own slum home. "I'll tell you all about it when I'm better," he whispered. But he did not get better for three days, and was still sitting propped up in bed, hardly able to breathe. Then "I'm so tired, sister," he said, "and so hungry," and straightway the breath of life troubled him no more.

A STRANGE BOY.

Another "heart-boy," Tommy Short, was better off than the last, for he had a father and mother who were altogether devoted to him. But with the unaccountable likes and dislikes that these patients get, he refused for many weeks before his death to have either of them sitting by him. He would greet them with a scowl and resolutely turn his back on them and shut his eyes. It was pitiable to see the poor mother creeping up behind the curtains to look at him, for every time he saw her he would cry "Go home! Go away home." The only time he would tolerate her presence was when he was lifted into a large cushioned chair, nursing a brilliant bulls-eye lantern, his chief treasure, and his mother was allowed to wheel him round and round the ward. He was only five years old, but nothing we could say to him had the least effect on his extraordinary stubbornness; and it was all the more odd as he had a terror of being left alone after the lights were turned down, yet he always drove his father and mother away, who would have been thankful to sit with him.

"A CLEAN LADY."

But the memories come thick and fast as the worn leaves are turned over, and one wonders where the boys are gone, and whether for good or ill, that used to crowd after tea to sit all piled up on the bed nearest the firelight while sister read them wonderful stories out of Grimm and Andersen. One is thankful to remember, at anyrate, that these little lads, out of very poor and often wretched homes, knew what it was to be tended and loved for at least a few days of their troubled childhood. Sometimes, one believes, the children appreciate their surroundings. A tiny little Jackie remarked the other day, "All de nice ladies dot in dis hospital!" and a small scrap of a visitor was seen admiringly gazing at a probationer in her Sunday cap, till at last she burst forth, "What a clean lady!"

### My First Experience in a Boys' School.

BY THE "NURSE MATRON."

I have had various kinds of nursing to do during my nursing career, but the work in which I am now engaged differs very much from any other.

### The School.

The school that I am in at present is one where only delicate boys are received, and the number of boys is limited, as we have only accommodation for about 30. It is situated in one of the most healthy parts of the South of England, stands very high, and is, in consequence, a most invigorating place, the air being fresh and bracing. The view is exceedingly pretty everywhere, but more especially on the north and east sides of the house. The gardens and fields cover an area of about 16 acres and are kept in very good order. There is every variety of vegetable in the gardens, also plenty of fruit.

### The Boys.

The ages of the boys vary from 17 to nine, the average age being about 13. They are very well and happy here, and great care is bestowed on them. Being delicate, no work is done before breakfast, which we have at 7.45 A.M., and during the day the order is so arranged that they are never long enough at any one lesson or game to get overtired.

### Recreation and Games.

During the winter months they spend some of their recreation time in wood-carving and in the carpenter's shop, and they also play football, hockey, and golf, and paper-chases are usually arranged for one day in each week up till the end of February. In the summer months there is cricket, lawn-tennis, and croquet. There are two large grass fields and a beautifully kept lawn for these games. The boys always have a run, if possible, in the fresh air during recreation hours, unless the weather is really too bad. If such should be the case, there is plenty to amuse them indoors in the way of billiards (there are two tables—a smaller one on which the juniors practise, and a larger one for the seniors), chess, draughts, halma, cards, etc.

### Treatment.

So far, I am thankful to say, I have had no illness among the boys, but only little minor ailments, such as headaches, colds, sprains, suppurating fingers, etc. My medicine chest, therefore, does not contain very much, though I generally keep the following in stock: Pure carbolic acid, boric acid powder, ointment, vaseline, lanoline, calomel, pil. rhei. co., M.M. c̄. M.S.; pulv. glycyrrhizæ co., ammoniated quinine, linseed oil, lint, bandages and strapping. The principal treatment has, therefore, been "preventive treatment," which I consider a most interesting branch of nursing. In the hospital we have no opportunity for this, as the patients, as a rule, come in so late; and the same applies to private nursing, for a nurse is, as a rule, only telegraphed for when the patient is very ill, but here it is quite different. By daily careful supervision and by watching the boys we soon detect the least symptom of anything abnormal, and, as a rule, the thermometer will decide for me, for I look upon it as being my "right hand," though some people put no faith in it. A small dose given statim will often set a boy right, and, as in the case of clothes, "A stitch in time saves nine," so in preventive treatment, "A little dose may save an illness." The regular cold baths which the boys have daily early in the morning and after a game or paper-chase in the afternoon, help to strengthen their constitutions. Some boys had never had them when they came here, but I feel certain they are all the better for them. The boys often tease me and say, "Oh, Nurse, wouldn't you like a real good case?" and then shortly after they will come and say they have a "temperature, headache, and feel ill." But I see through them now, and know that they are no exception to the rule that "boys will be boys all the world over."

### Presentations.

Johannesburg Hospital.—A pleasant and interesting function took place at the Johannesburg Hospital on July 27th, when the lady superintendent entertained Sister Waller to a complimentary dinner on the occasion of her leaving that institution in order to take up the duties of matron at the new Primrose Hospital. All the sisters in charge were present, and the menu of the dinner included "Saddle of venison à la Waller" and "Bradford pudding." Mrs. Magill, in making the presentation of the handsome travelling clock from the staff, gave a *résumé* of Miss Waller's career since she had trained her at Bradford Royal Infirmary until the present day. A very enjoyable evening was spent, and the kindness of the matron was greatly appreciated.

### The Nurses' Bookshelf.

The Kingdom of Twilight. By Forrest Reid. (First Novel Library. Fisher Unwin. 6s.)

As a first book this has much to recommend it, and it is full of promise for the future. The character of Willie Trevellyan, a poet, dreamer, and generally misunderstood youth, is so sympathetically drawn that in following its development one forgets the absence of plot. As a whole it is a sad story; but some relief is given by the introduction of Mrs. Gower, described as a woman "whose extremely wide experience and knowledge of the world had given to her views and conversation a certain flavour of perhaps over emphasised sympathy with the rogues and vagabonds of life, but who also treated other people's lack of a similar complacency with unfailing good humour." Her niece Eva, whose friendship for Trevellyan is the most charming part of his history, and his entanglement later with a modern Circe, are the two influences that count in it. "The Kingdom of Twilight" is a book which conveys its own lesson—one which parents with children of misunderstood temperaments will be wise to reflect upon.

### TRAVEL NOTES AND QUERIES.

By our Travel Correspondent.

Holidays in September (Erin).—There is very little chance of the house at Brixham being still available, but I will give you the address if you send me a stamped and addressed envelope. Over and over again I beg my correspondents to tell me what sum they can afford to pay; such questions as yours are too vague. What do you mean by "moderate terms"? Your idea of the meaning of the word moderate may be quite different from mine. No one sees correspondents' letters except myself, so why not be frank? It would save me infinite trouble and I could help you much more satisfactorily. We do not care to send addresses about (which proceeding takes time and trouble) and then find because of total ignorance as to our correspondent's needs that we have worked idly and been of no help. Tell me how much you can comfortably afford and I think I can tell you of suitable places. Would you like the Isle of Man, and would you like to go abroad, rather than spend your holidays in England?

### Everybody's Opinion.

[Correspondence on all subjects is invited, but we cannot in any way be responsible for the opinions expressed by our correspondents. No communication can be entertained if the name and address of the correspondent are not given as a guarantee of good faith, but not necessarily for publication. All correspondents should write on one side of the paper only.]

### THE NEW RULES AT ST. ANDREW'S HOUSE CLUB.

A MEMBER of St. Andrew's House Club writes:—Through the medium of your valuable paper will you kindly make known your opinion of the enclosed prospectus? I for one consider this "club" might be termed "a private hotel." To call it "a home for nurses" is a farce. The exorbitant charges are out of all proportion to any advantage the resident nurse members have—that is my experience. This proprietor, a few years ago, allowed us to believe that her sole aim and object in opening St. Andrew's House Club was to benefit nurses between their cases. It was to pay its own expenses, of course; that was all that would be expected. We were, however, soon disillusioned. Steadily charges for one thing and another have cropped up or increased. The food is neither better nor worse than in other houses where nurses are obliged to live. As for the restaurant I have always been under the impression that food asked for between meals could not be relied upon being served, unless a journey had to be taken, when the favour would be granted. The electric lift, for the use of which members are charged, has always appeared to be principally for the servants' benefit between 7 A.M. and 10.30 P.M. The extra charge for baths may not be demurred to if the proprietor will see to their being made a little more inviting.

### RED TAPE IN THE WARDS.

"BET" writes: "Those who look on see the most" is very true, and the "looker on" who saw such "red tape in the wards" must have seen more than there generally is to be seen. The arguments she uses are extremely weak, especially when she tells us that the babies' doubles must not be dried by the fire for fear of spoiling the look of the ward. Why not also add, nurses must not wash the doubles in the ward for fear of making the ward look like a laundry? I would ask, why should these things be done in the ward when they can be done elsewhere?—for instance, in the bath-room, which generally adjoins the babies' ward. When the patient was rebuked when the doctor was expected, did he not report the matter to the doctor when he arrived? He would have had every right to do so. A demand for a toothbrush of course would surprise one, but if the patient said "Nurse, will you give me a toothbrush?" no nurse would show surprise at the request. "Looker-on" says that "the hospitals to which she refers are excellent institutions." I say that they cannot be, or such things as she saw could not have taken place. Is it necessarily only when one is no longer young that one's sympathetic and critical faculties become fully awake? Is it possible to be taught sympathy? Without one's sympathetic nature was well developed surely one would not choose to be a nurse, and if in busiest moments the—poor chap—asks for a drink—well just give him one, and his "Thank you, nurse," will reward you for any remote risk you are running of being—sat upon. The nurse who reports "that awful No. 3 has been sick" would be corrected; but a nurse's life is very trying, and if sometimes she is a little impatient, after perhaps being on duty 12 or 13 hours, one should remember that she is a woman and is really trying to do her best. No one is perfect, so why look for perfection among nurses especially. Why not all try conscientiously to improve and to make our very best better?

"ONLOOKER" writes: Would you be kind enough to correct a misprint which appeared in my letter to THE HOSPITAL. I did not say "it is only fair *as* the writer of "Red Tape in the Ward," but "*to* the writer." The latter is quite unknown to me.

### Appointments.

[No charge is made for announcements under this head, and we are always glad to receive, and publish, appointments. The information, to insure accuracy, should be sent from the nurses themselves, and we cannot undertake to correct official announcements which may happen to be inaccurate. It is essential that in all cases the school of training should be given.]

BRISTOL UNION INFIRMARY, EASTVILLE.—Miss Helen Pirie has been appointed superintendent nurse. She was trained at King's Cross Isolation Hospital, Dundee, and the Crumpsall Infirmary, Manchester. She has since been sister at Crumpsall Infirmary. She holds the certificate of the London Obstetrical Society.

DEWSBURY HOSPITAL.—Miss Magdalen Cartwright has been appointed matron. She was trained at the Royal Alexandra Hospital, Rhyl, and the Royal Southern Hospital, Liverpool. She has since been charge nurse at the Children's Hospital and Toxteth Park Infirmary, Liverpool; ward sister at Birmingham City Fever Hospital; ward sister at Skipton Infectious Diseases Hospital. She is at present taking matron's holiday duties at Skipton Hospital.

FLORENCE NIGHTINGALE ISOLATION HOSPITAL, BURY.—Miss Kate Thomas has been appointed sister. She was trained at the Crumpsall Infirmary, Manchester, where she has since been sister. She holds the certificate of the London Obstetrical Society.

GAINSBOROUGH ISOLATION HOSPITAL.—Miss Valetta Shout has been appointed matron. She was trained at the Alexandra Hospital for Children, London, and at Addenbrookes Hospital, Cambridge. She has since been staff nurse and night superintendent (*locum tenens*) at the Sanatorium, Hull; charge nurse at the City Hospital, S. Liverpool; matron of the Sanatorium, Morecambe, and matron (*locum tenens*) of the Sanatorium, Lancaster.

HERTFORD AND WARE JOINT HOSPITAL.—Miss G. M. Philpott has been appointed charge nurse. She was trained at the St. William Hospital for Infectious Diseases, Rochester, where she was afterwards charge nurse.

QUEEN ALEXANDRA'S MILITARY NURSING SERVICE FOR INDIA.—Miss Marion Stephenson has been appointed nursing sister. She was trained at Mill Road Infirmary, Liverpool, has since been charge nurse at the South-Eastern Fever Hospital and on the staff of the London Association of Nurses, 123 New Bond Street. She is also a member of the Alexandra Nursing Service Reserve, and has served in South Africa and at the Military Hospitals at Norwich and Caterham.

REDHILL COTTAGE HOSPITAL.—Miss Nellie Louise Slaughter has been appointed staff nurse. She was trained at the Ipswich Hospital and at the London Hospital, Whitechapel, E.

STEPNEY UNION.—Miss Agnes M. Conner has been appointed assistant staff nurse. She was trained at Hackney Union Infirmary, and has since been charge nurse at the Central London Sick Asylum.

THOMPSON MEMORIAL HOSPITAL, LISBURN, IRELAND.—Nurse Bakewell has been appointed night superintendent. She was trained at the Victoria Infirmary, Norwich; the Fever Hospital, Manchester; and the Fulham District and Maternity Home. She was charge nurse at the North Riding Infirmary and the Cancer Hospital, and has undertaken temporary sister's duty.

WALLINGFORD UNION INFIRMARY.—Miss Martha Louise Mancer has been appointed superintendent nurse. She was trained at Steyning Union Infirmary, where she was afterwards charge nurse, and she has since been charge nurse at Prescot Union Infirmary and superintendent nurse at Christchurch Union Infirmary.

### Echoes from the Outside World.

### Movements of Royalty.

The King enjoyed fine weather at Marienbad up to Monday, but on that day it became cold and showery. Notwithstanding the unpleasant change his Majesty took his customary exercise. During the day he visited the Austro-Hungarian Ambassador to Great Britain. The King is expected to arrive in London about September 4th. On Saturday morning Princess Christian, with Princess Victoria of Schleswig-Holstein, left England in the *Walmer Castle* for South Africa in order to see the grave of Prince Christian Victor in Pretoria. They will land at Cape Town and proceed up country by railway.

### The Siege of Port Arthur.

The Mikado's summons to the Russian commander to surrender Port Arthur met with a flat refusal from General Stoessel, and his invitation to send noncombatants to a place of safety was not accepted. When the terms—which were that the garrison should march out with all the honours of war and join General Kuropatkin, and that the Russian ships in the harbour should be handed over to the Japanese—were submitted to General Stoessel, he broke out into invective, but treated the Japanese officer with courtesy. From Sunday until Wednesday last week a great battle raged around Port Arthur. The Japanese are said to have sustained enormous losses, but to have gained important advantages of position. The strength of the Russian garrison is estimated at 23,000 men, who are holding a line 12 miles long. The various military and naval attachés have left. According to telegrams from Chifu the Japanese have received large reinforcements, and have captured several positions, but been unable to hold them in the face of the fire from the Russian batteries.

### The Two Sea Fights.

In the official report of the Russian admiral commanding the Port Arthur and Vladivostock squadrons respecting the two sea fights on August 10th and 14th, it is stated that the damage sustained by the cruisers was very great; five officers were killed and many wounded, and 135 sailors were killed and 307 wounded. Admiral Kamimura in his report of the engagement says that he manœuvred so as to keep his back to the sun, whereby his gunners had a great advantage. He bears the highest testimony to the splendid gallantry of the Russians; also to their care of the wounded, many of whom, some without arms or legs, were placed on planks and lowered into the sea before the *Rurik* sank.

### The British Mission to Thibet.

It appears from a despatch from Lhasa, dated August 14th, that the Thibetans have had enough of fighting. The Amban has assured Colonel Younghusband that the common people have been impressed by the humanity with which their wounded have been treated, and the punctuality with which payment has been made for requisitioned supplies. With our advent at Lhasa, the monks themselves have largely lost their reason for hostility. They are aware that the monasteries and temples are at the mercy of the British, and their frequent assertion of a desire for peace is believed to be entirely genuine.

### Lord Minto and the Plains of Abraham.

Lord Minto, the retiring Governor-General of Canada, in a speech which he delivered at Quebec on Friday, told his hearers that he loved to look upon the Plains of Abraham, hallowed as they were by time and rich in historic association. Vandalism, he continued, seemed to be inserting its wedge, and the glorious battlefield was in danger of being sacrificed. Improvements were well enough in their way, but there was such a thing as too ruthlessly destroying old landmarks. He assured his hearers that he had no sympathy with the vandalism to which he referred. The remarks of Lord Minto constitute a condemnation of the action of the Canadian Government in handing over a portion of the battlefield for the erection of an unsightly factory.

### New Black Rod.

The King has appointed Admiral Sir Henry Frederick Stephenson, G.C.V.O., K.C.B., to be Gentleman Usher of the Black Rod in the room of General Sir Michael Biddulph, G.C.B., deceased. The office of Black Rod is in the personal gift of His Majesty, who in bestowing it upon a distinguished officer in the Navy has followed precedent, the last holder of the post being an eminent officer in the Army. Sir Henry Stephenson, who is first and principal Naval A.D.C. and Extra Equerry to the King, and was formerly Naval A.D.C. to Queen Victoria and Equerry to the Prince of Wales, was born in 1842. He served in the Crimean War, the China Expedition of 1857, the Indian Mutiny, Canada, Egypt, and as captain of the *Discovery* in the Arctic Expedition of 1875-76, and commanded successively the Pacific and Channel Squadrons. Last December he married the Hon. Charlotte Keppel, widow of Colonel W. H. A. Keppel, and sister of Lord Saltoun.

### The Intellectual Power of the Sexes.

At the meeting of the British Association at Cambridge, over which the Prime Minister presided, a remarkable paper was read by the Director of Municipal Statistics at Budapest, in which he instituted a statistical comparison of the intellectual power of the two sexes. His material was taken from observations during the past 27 years in the schools of Budapest, where a special report of each pupil's progress had been sent to his office. These reports related at first to elementary schools, where the ages ranged from six to 12 years; also to the higher elementary schools, where the ages ranged from 10 to 12 years; and, further, to grammar schools. The test as to the general progress in the elementary schools was the number of children who had to repeat their first year's work instead of passing on to the next standard; and out of 412,758 boys 16·8 per cent. repeated it, and out of 350,382 girls 15·8 per cent. In the higher elementary schools, out of 14,201 boys 6·2 per cent. and out of 20·588 girls 2·2 per cent. had to repeat their work. The results were thus all in favour of the feminine sex, but they related only to children. With regard to later life Dr. De Körösy thinks that as not only in sciences, but in poetry and in arts also—except the stage—the greatest work of human progress has been accomplished by men, there is a turning-point at which the intellectual activity of women seems to be arrested.

### Attempts to Swim across the English Channel.

On Saturday night Montague Holbein made a fifth attempt to swim across the English Channel, but was again unsuccessful, his failure being due to the fact that he swallowed salt water, which caused sickness and other trouble. When he had been swimming for 10 hours and 20 minutes he abandoned the task and was assisted into one of the accompanying rowing boats. Haggerty, diving from the end of the Admiralty Pier on Saturday night, succeeded in getting about two miles from the shore, and then, being seized with cramp, was nearly drowned before he was pulled into a boat. Greasely, who entered the sea from the same pier, gave up when he had been swimming less than an hour.

### Notes and Queries.

**FOR REGULATIONS SEE PAGE 143.**

*Electrolysis.*

(159) I have a slight growth of hair on my upper lip; do you advise electrolysis? Can you recommend anyone to perform it? What would be the probable cost, and is the process painful?—*Gwynnie.*

Electrolysis is the only satisfactory treatment known. The process is tedious and expensive, but not necessarily painful. Inquire for particulars as to obtaining treatment at the Manchester and Salford Hospital for Skin Diseases, Quay Street.

*Maternity Club.*

(160) Can you give me any idea how to form and carry on a maternity club? Some of the people in this district are very poor and I thought it possible that a club could be formed into which the women might pay small weekly contributions in order that they might have sufficient to pay doctor and nurse, etc.—*District Nurse.*

You will obtain some useful information in "District Nursing on a Provident Basis," by Jamieson B. Hurry, M.D. It can be obtained from the Scientific Press.

*Myopia.*

(161) Will you kindly tell me if myopia is curable? I have been advised to wear glasses and I want to know if there is any advantage in wearing them continually or simply when required? Will you kindly let me know what hospital, if any, treats the defect?—*T. M. W.*

Myopia is not curable. You had better consult a competent oculist or attend any of the larger general hospitals or ophthalmic hospitals.

*Matron.*

(162) Will you kindly tell me what training is necessary to qualify a nurse as matron in a fever hospital? Is training in general nursing essential?—*Nurse B.*

In the fever hospitals under the Metropolitan Asylums Board even charge nurses are required to hold a certificate of three years' training in general nursing. Special training in a fever hospital is also necessary.

*Books.*

(163) Will the books on nursing by Alexander Miles, J. K. Watson, or Isabella Hampton give information bearing upon surgical nursing, instruments, splints, etc., with directions for "Gum and Chalk," "Bavarian," etc.?—*Delta.*

Miles' "Surgical Ward Work" for instruments and "Surgical Bandaging and Dressings," by Dr. Johnson Smith, for "general surgical nursing" would give you all you want.

*Advice.*

(164) I am a certificated obstetric nurse, midwife, and masseuse; I am 26 years old, and get plenty of work, generally earning £2 2s a week. Would it improve my position if I took a three years' certificate in general nursing? It seems a pity to leave my present work.—*Forethought.*

If you have a good connection and large experience, would it be worth your while to give up three years' practice?

*Home.*

(165) Will you kindly tell me of a superior home or institution where a little girl, aged two months, who has lost her sight could be received? Her parents are willing to pay for her.—*Nurse E.*

Write and ask the Secretary of the British and Foreign Blind Association, 206 Great Portland Street, W.

*Hospital Training.*

(166) Will you kindly tell me where I, at 25 years of age, could be trained, and receive a three years' certificate in general nursing?—*Selborne.*

See "The Nursing Profession: How and Where to Train."

**Important Nursing Text-books.**

"The Nursing Profession: How and Where to Train." 2s. net; 2s. 4d. post free.

"The Nurses' Dictionary." By Honnor Morten. 2s. post free.

"Nursing: a Complete Text-book of Medical, Surgical, and Monthly Nursing." By Percy Lewis, M.D. 3s. 6d., post free.

"Elementary Physiology for Nurses." By C. F. Marshall, M.D. 2s. post free.

"A Complete Handbook of Midwifery for Nurses." By J. K. Watson, M.D. 6s. net; 6s. 4d. post free.

The above works are obtainable of all booksellers, or direct from The Scientific Press, Limited, 28 & 29 Southampton Street, Strand, London, W.C.

### For Reading to the Sick.

WAITING IN TRUST.

Unseen by thee, His helpful presence fills
The herbless plain and bleak unwatered way;
That in due season shall the mountain rills
Murmur their music to the broadening day:
And Joy shall crown the hill-tops with her smile,
And Love shall walk the meadow-paths awhile.

For He still passeth where the ripe corn sways
In the full glory of the harvest-field;
The echo of His voice all fear allays—
Tells the sure promise of a seven-fold yield:
The promise that, as days their hours unroll,
His presence is the Sabbath of the soul.

So shall He give to thee the ripened ears,
And thou shalt eat thereof, and true life live;
And all the labour of the lonely years,
Safe-garnered in His barns, rich treasure give:
And thou shalt surely know, with heart elate,
Blessed are they who, trusting, work and wait.

*W. G. Kingsland.*

The circumstances of her life remained unchanged, but her soul was kept in perfect peace in the midst of them. And the secret she found so effectual in her outward affairs, she found to be still more effectual in her inward ones, which were in truth even more utterly unmanageable. She abandoned her whole self to the Lord, with all that she was and all that she had; and, believing that He took that which she had committed to Him, she ceased to fret and worry, and her life became all sunshine in the gladness of belonging to Him.—*H. W. S.*

"Let not your heart be troubled." Cast aside, that is every thought of self, which can only bring estrangement, defeat, humiliation: admit no misgivings of personal frailty: trust to no presumptuous promptings of personal confidence: look without you and not within: look to the Father whom I came to declare: look to Me, whom you have learnt to know. So you will perceive that I go to make ready for you a place in a heavenly home: that I go for a while in order that I may bring you to Me for ever; "and whither I go, ye know the way."—*B. F. Westcott.*

Our Lord Jesus Christ would have us learn that Eternity is not so much a future state, to which the stream of time is bearing us on, as a present but unseen state, and that in fact we have all now entered on Eternity. It is around us now, and we can none of us get out of it. We have begun and must go on living for ever. God is not a being dimly thought of, separated from us above our heads; but not far from any one of us, the Maker and Ruler, in whom we live and move and have our being.—*R. W. Sibthorp.*

God doth not bid thee wait,
To disappoint at last;
A golden promise fair and great
In precept-mould is cast.
Soon shall the morning gild
The dark horizon-rim.
Thy heart's desire shall be fulfilled,
Wait patiently for Him.

*F. R. Havergal.*

